# Exploring the Sustainable Use Strategy of Scarce Water Resources for Rural Revitalization in Yanchi County from Arid Region of Northwest China

**DOI:** 10.3390/ijerph192316347

**Published:** 2022-12-06

**Authors:** Yongsheng Wang, Xiao Cui, Xinrong Zhang, Qi Wen

**Affiliations:** 1 Key Laboratory of Regional Sustainable Development Modeling, Institute of Geographic Sciences and Natural Resources Research, Chinese Academy of Sciences, Beijing 100101, China; 2 University of Chinese Academy of Sciences, Beijing 100049, China; 3 School of Earth Science and Resources, Chang’an University, Xi’an 710054, China; 4 School of Geography and Planning, Ningxia University, Yinchuan 750021, China

**Keywords:** rural revitalization, climate change, agricultural water demand, water sustainable utilization

## Abstract

Water scarcity limits the coordination between economic development and ecological protection of arid regions. This study presented the consumption pattern and future challenges for water resources and proposed sustainable use strategies for water security in Yanchi county from the arid region of northwest China. Our results showed that water withdrawals were close to the total available water resources. Agriculture consumed about 84.72% of the total water supply. Agricultural water use was influenced by breeding stock, rural per capita net income and effective irrigation area. Estimation of agricultural water demand was about 6582.20 × 10^4^ m^3^ under the rural revitalization scenario. Limited water supply and increased water demand pose challenges and impediments for rural revitalization and water security in Yanchi county. Water sustainable utilization can be achieved by increasing water supply from unconventional water resources and improving water use efficiency with governmental management. These findings may help policymakers to develop sustainable water use strategies during rural revitalization in arid regions.

## 1. Introduction 

Water resources are fundamental for food production, the preservation of ecosystems, the sustenance of communities, and the survival of life itself [1]. Water is crucial for poverty alleviation and economic development in the World [2]. At the regional level, freshwater competition between countries leads to dangerous trans-boundary tensions and conflicts everywhere in the World [3]. At the country level, economic sectors such as agriculture, industries, services and households compete for available freshwater resources. Water shortages have become an urgent problem threating human society. For example, water shortage disasters resulted in the disappearance of China’s Loulan Civilization [4]. Water poverty has a significant correlation with regional economic poverty [5]. Therefore, water has been placed at the core of Sustainable Development Goals [6]. There are 15 Sustainable Development Goals that relate directly or indirectly to water.

Human activities and climate change have placed more pressure on global water security [7]. Continuing population growth, expanding irrigated croplands, and changing dietary patterns have undoubtedly caused the growing demand for water [8,9]. Many rivers cannot satisfy the quantity and quality of local water demands [10]. Over-exploitation of groundwater for agricultural irrigation, human and livestock drinking, and industrial development cause groundwater shrinkage and surface subsidence [11,12]. In addition, worsening climate crises have been inducing negative effects on global agricultural production [13]. Climate change led to a 50% reduction of rain-dependent agriculture in 2020. About two-thirds of the World’s population will be living under water stress in 2025 [1]. Especially in arid and semi-arid regions of the globe, such as southern Europe, western Asia, Sahara region, and northwest China, persistent water issues, such as scarcity, drought, depletion of aquifers and conflicts, are key hazards for local inhabitants. Water crises will become more frequent and serious under ongoing economic development. Climate change is projected to increase water deficits [14]. Serious water-related problems will be difficult to restrain if non-sustainable water management practices are continued.

In China, per capita water resources are only one-quarter of the global average, while water consumption per unit of the gross domestic product is three times the world average [15]. Over three-fourths of China’s 669 cities face water shortage problems [16]. In 2050, per capita water resources will decline to about 1700 m^3^, approaching the threshold of severe water scarcity [17]. In addition, over half of the population will be affected by water pollution and its regional inequalities [18]. The Yellow River is known as the “Mother River” and cradle of “Chinese Civilization” while facing water deficiency and water pollution. The North China Plain will be uninhabitable due to the deadly wave caused by climate change, artificial irrigation, and water pollution [19]. 

The northwest region accounts for 35.9% of China’s total land areas, while water resources only account for 5% of the whole country. The annual rainfall of most areas is less than 300 mm, with 70% of precipitation falling in the form of storms [20]. Lower precipitation and higher atmospheric evaporation have led to long-term and severe water scarcity which make northwest China face the discrepancy between water supply and demand. Lower water use efficiency and unreasonable water use structures in northwest China has led to a water resources exploration and utilization rate that is 2.65 times higher than that of the whole country [21]. A geographical mismatch between farmland and water availability has given rise to widespread unsustainable agricultural expansion in China’s dry areas [22]. 

Water shortages can directly decrease agricultural productivity and indirectly affect both rural incomes and food security [23]. Low amounts or a lack of access to safe water are among the most important factors restricting poverty alleviation and economic development. In water shortage and land degradation regions, local residents have no alternative means of earning a sustainable living other than previous unsustainable activities [3], causing a vicious circle of environment degradation and rural poverty [20,24]. Economic poverty was significantly related to water poverty in rural China from 1997 to 2019 [5]. About 20.44% of China’s poverty-stricken counties were restricted by water resources [25]. Recurrent droughts restrict farmers’ abilities to overcome chronic poverty [26]. China’s government has established many water policies and measures to alleviate rural poverty, such as soil and water conservation [27], degraded land consolidation and agricultural development [28]. 

In 2017, China’s rural revitalization received more attention since Liu and Li [29] advocated revitalizing the World’s countryside. The rural revitalization strategy has become the main agenda of China’s governmental work on agriculture, rural areas and rural residents since 2018. China’s rural revitalization strategy will consolidate and promote rural development after targeted poverty alleviation. However, the water crisis is always the biggest challenge during rural development in China’s arid and semi-arid regions, and this condition will be further aggravated due to the interactions between rural revitalization, climate change and urbanization. 

In 2019, China’s Ministry of Water Resources released the guideline on water conservancy work in the strategic planning for rural revitalization. Thus, developed industry, improved living standards, and concerned ecological construction should respect the carrying capacity of the water resources in arid and semi-arid regions. Many approaches to long-term water planning and management have been explored by experts and organizations in arid and semi-arid regions. Appropriate strategies and guidelines for water conservation and supply have been suggested from the perspectives of technologies, projects, policies and laws. However, it is difficult to increase water supply because of the resource endowment constraints in many arid and semi-arid regions. Sustainable measures are urgently needed to cope with the escalating water demands from rapid social-economic development and the competition for water among different water-demanding sectors [30]. Therefore, identifying the actual needs and achievable methods are essential to bridging the water gap [31]. Yanchi county from arid region of northwest China is a typical region with scarce water resources depending on the diversion of the Yellow River. Given rural revitalization and urbanization, the gap between water demand and supply will be increased. Sustainable water management strategies will appeal to local policymakers addressing water deficiency. Therefore, our objectives were to (1) reveal the water consumption pattern of Yanchi county; (2) identify the influence factors of agricultural water consumption based on the framework between water utilization and rural economic development; and (3) predict agricultural water demand and provide a sustainable use strategy of scarce water resources for rural revitalization. The results will provide sustainable water management theories and practices for poor countries in arid and semi-arid regions.

## 2. Materials and Methods

### 2.1. Site Description

**Figure 1:**
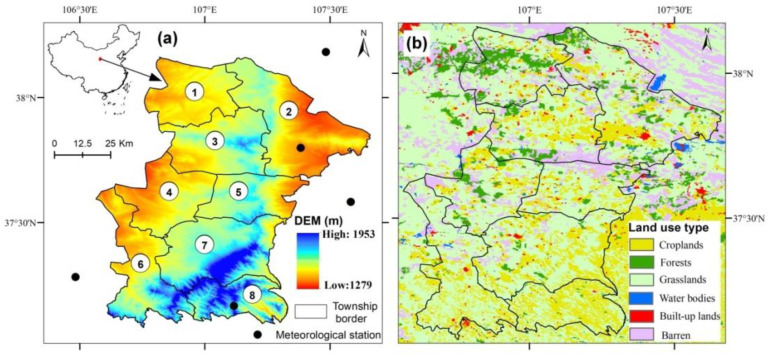
The terrain (**a**) and land use types (**b**) of the study area. Note: Yanchi county includes eight administrative towns, which are Gaoshawo (①), Huamachi (②), Wanglejing (③), Fengjigou (④), Qingshan (⑤), Huianpu(⑥), Dashuikeng (⑦), and Mahuangshan (⑧), respectively.

Yanchi county is located in the transitional zone at the border of Ningxia, Inner Mongolia, Shaanxi and Gansu provinces. Yanchi has a vast area of 8661 km^2^ and a sparse population of 172,600, involving eight towns and 102 administrative villages. The elevation decreases from 1953 m a.s.l in south region to 1279 m a.s.l in north region (Figure 1a). The dominated land use type is grassland and cropland, accounting for 47.4% and 23.3% of the county’s total area, respectively (Figure 1b). The mean annual precipitation and temperature is 290 mm and 7.8 °C, respectively. In 2018, Yanchi became the first county in Ningxia Hui Autonomous Region to be delisted from China’s poverty county roster. It also had been recognized as the first demonstration of achieving an effective connection between rural revitalization and targeted poverty alleviation in northwestern China. 

### 2.2. Water Use and Rural Economy

**Figure 2:**
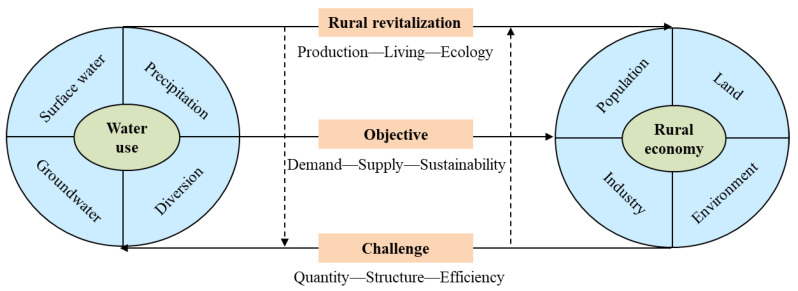
Framework between water use and rural economy under the context of rural revitalization.

Surface water, groundwater, precipitation and diversion water are the basic sources of water. The carrying capacity of water resources involving quantity and quality is indispensable to human activity and thriving life. Water should be used as its capacity permits, water determines population, land production, industry development and living environments (Figure 2). 

Thriving industry, pleasant living environments and prosperity are the three of the five goals of China’s rural revitalization strategy. The challenges presented by increasing water quantity demand, changing water use structure, and comparable lower water use efficiency need to be highlighted during rural revitalization (Figure 2). Increased water use can be explained from by the following aspects. First, more and more high-standard farmland and competitive agricultural industry will be established to support rural economic development. Clean and safe tap water supplies in rural areas make cooking, washing, and bathing more convenient than before. A beautiful countryside will attract more and more tourists from urban areas. Improved production and living conditions, and booming rural tourism significantly increase water demand. In addition, water is the foundation of rural landscape construction and is a booster for ecological environmental improvement. Rural construction and development exacerbate competition for water demand related to production, living and ecology, causing increases, and even shortages in water use [32]. 

Especially in arid and semi-arid regions, efficient water management and sustainable use strategies are the lifeblood of rural revitalization. Industrial layout, infrastructure development, land use and public service will be given priority when carrying out rural revitalization. Finding the balance between water demand and supply and explore the sustainable use strategy is crucial to achieve rural revitalization and a comprehensive well-off society in the future.

### 2.3. Influence Factor Analysis for Agricultural Water Use

#### 2.3.1. Evaluation Indicator System

The generalized water resources complex system is related to the social-economic system and ecological environment system (Figure 2). The agricultural sector plays the dominant role in water use in China [33]. Agricultural water demand will increase along with the increased population, rising incomes and changing dietary patterns [34]. In addition, climate change has become the major challenge for agricultural sustainable development [35]. Yanchi is a typical agricultural-dependent county with abundant land and scarce water resources. According to previous studies [36,37] and a framework between water use and rural economy (Figure 2) and the actual water consumption situation of Yanchi county, an evaluation indicator system of agricultural water use was constructed from the perspectives of population pressure, farmland use, industry development and environmental change (Table 1).

#### 2.3.2. Influence Factor Analysis

Influence factors of agricultural water use were analyzed using the model GeoDetector [38]. The relative importance of the influence factor (X) to the agricultural water use (Y) can be quantitatively described using the *q*-statistic [38] in the following equation. The effects of two influence factors can be calculated by comparing the *q* values between the interaction and single [39] using the sector of interaction detector:

(1) $$q=1\;-\frac{1}{{N\sigma }^{2}}\overset{L}{\underset{h=1}{\sum }}N_{h}\sigma_{h}^{2}$$

where h is the hierarchy of factor *X*, *N* means the number of county units, and $\sigma_{h}^{2}$ represents the variance value of variable *Y*.

The interaction detection was used to test the effect of different influence factors on the agricultural water use. q(X1) and q(X2) were calculated to represent the explanatory forces of the factors of X1 and X2, respectively. q(X1∩X2) was calculated to represent the interaction effects of X1 and X2 on agricultural water use. The interaction between two independent variables can be divided into five categories [39] by comparing q(X1∩X2) with q(X1) and q(X2) (Table 2).

### 2.4. Agricultural Water Demand Estimation

The total agricultural water demand (TAWD) in different irrigation region is calculated using Expression (2) and Expression (3).

(2) $$\mathrm{TAWD}={\mathrm{AWD}}_{y}+{\mathrm{AWD}}_{\mathrm{ys}}+{\mathrm{AWD}}_{w}$$

(3) $$\mathrm{AWD}=\underset{i}{\sum }\left(A_{i}\times Q_{i}\right)/\mathrm{WE}\;$$

where ${\mathrm{AWD}}_{y}$, ${\mathrm{AWD}}_{\mathrm{ys}}$,${\;\mathrm{AWD}}_{w}$ represent the actual agricultural water demand in the Yellow River irrigation district, Yellow River supplement irrigation district and well irrigation district, respectively. $A_{i}$ and $Q_{i}$ represent the planting area and quota of different crop, respectively. WE represent the water use coefficients in different irrigation districts.

### 2.5. Data Sources 

Water resource data, including water withdrawal and consumption, effective irrigation area, and a water use coefficient were collected from the Bureau of Water Supplies of Yanchi county. Breeding stock, cultivated land output benefit and planting agricultural structural adjustment data in the context of rural revitalization were collected from the Bureau of Agriculture and Rural Affairs of Yanchi county. Irrigation quotas were collected from Agricultural Irrigation quota in Ningxia (http://www.jsgg.com.cn/Files/PictureDocument/20181109184843850485281097.pdf, accessed on 12 December 2021). Precipitation and temperature data were collected from the Chinese Meteorological Administration (http://data.cma.cn/). These data have undergone a rigorous quality inspection and control by the Chinese Meteorological Administration. We interpolated gauge-based data into 0.05° × 0.05° gridded data using professional meteorological interpolation software, namely the Anusplin [40]. The other data were collected from Yanchi county population census summary, township government work report and the department of Yanchi county. In addition, a village investigation was conducted in December 2018 and March 2019.

## 3. Results

### 3.1. Water Withdrawal and Consumption

**Figure 3:**
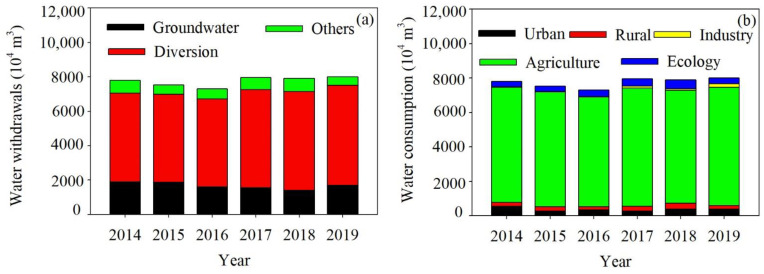
Water withdrawals from different sources (**a**); and consumption in different sectors (**b**).

Water withdrawals decreased from 7802 × 10^4^ m^3^ in 2014 to 7297 × 10^4^ m^3^ in 2016, and then increased to 7998 × 10^4^ m^3^ in 2019. A decreased trend was found in groundwater withdrawals while the Yellow River water withdrawals significantly increased from 5148 × 10^4^ m^3^ in 2014 to 5800 × 10^4^ m^3^ in 2019. The Yellow River water diversion contributed 65.98–72.61% to the water withdrawals of Yanchi county (Figure 3a).

As the highest water use sector, agriculture accounted for 83.05–88.47% of the total water consumption. Industrial water consumption increased up to 5.04 times from 33.77 × 10^4^ m^3^ in 2014 to 204.01 × 10^4^ m^3^ in 2019. Significant interannual variations were found in the water consumption from urban, rural and ecological sectors (Figure 3b). 

### 3.2. Geodetection of Agricultural Water Use

**Figure 4:**
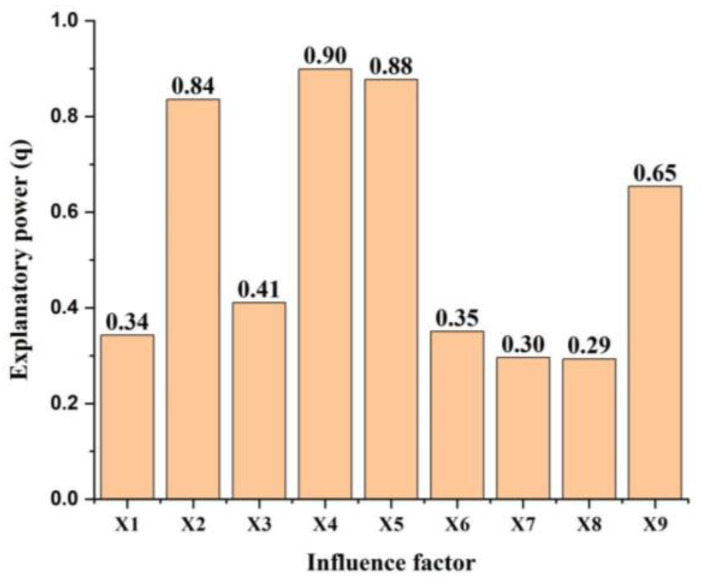
Influence factors of agricultural water use in Yanchi county.

Breeding stock (X4), rural per capita net income(X5) and the effective irrigation area (X24) were the main influence factors of agricultural water use. The effect of actual evapotranspiration (X9) on agricultural water use was also significant, being the q value of 0.65. Total population (X1), precipitation (X7), temperature (X8) and proportion of the primary industry (X6) had few effects on agricultural water use (Figure 4).

**Figure 5:**
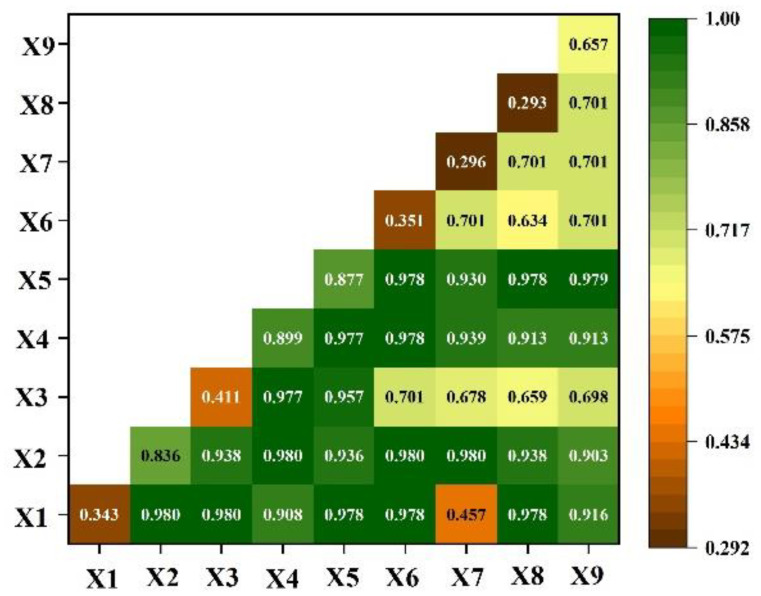
Interaction detecting analysis of influence factors for agricultural water use in Yanchi county.

A better explanation was found between the two-factor interaction and single factor. Breeding stock (X4), rural per capita net income(X5) and effective irrigation area (X2) had a higher interaction explanation q value of more than 0.9 compared with other factors. Interaction q value between total population (X1) and precipitation (X7) was the lowest (Figure 5).

### 3.3. Agricultural Water Demand Prediction 

Total agricultural water demand under rural revitalization is 6582.20 × 10^4^ m^3^. The Yellow River Irrigation District has the highest predictions of agricultural water demand of 3391.00 × 10^4^ m^3^. The Yellow River Supplement Irrigation District area is 9807 ha with a water demand of 2080.53 × 10^4^ m^3^. The Well Irrigation District has the lowest area and highest water utilization coefficient, resulting in the lowest water demand of 1110.67 × 10^4^ m^3^ (Table 3).

## 4. Discussion

### 4.1. Challenges between Water Supply and Demand

A sharp contradiction between limited water supply and increased water demand poses challenges and impediments for achieving water security under a rural revitalization scenario in Yanchi county. The per capita water resources of Yanchi county are lower than one-fifth of the national average, and one-third of Ningxia’s average [41]. Total water withdrawal in Yanchi county was approximately equal to the threshold value. Total available water resources are 8000 × 10^4^ m^3^, largely depending on the Yellow River Diversion with value of 5800 × 10^4^ m^3^ and groundwater with value of 2000 × 10^4^ m^3^. However, total water withdrawals, Yellow River water Diversion and groundwater exploitation has reached its limit since 2014 (Figure 3a). The Yellow River diversion quota is fixed and will be difficult to increase in the future. Although, groundwater has played an important role in water supply (Figure 3a). The poor quality of groundwater has little potential for exploitation and threatens the health of local residents [42]. According to the IPCC Sixth Assessment Report, climate change has exacerbated the global hydrological cycle, with negative impacts on water security [43]. Increased irrigation water consumption will occur in Asia and western United States based on the ensembled simulation results from eight general circulation models and seven global hydrological models [44]. In the arid and semi-arid regions of China, it is exciting that the average precipitation and runoff will increase by 17.5% and 9.1% in the 2030s, respectively, compared with the 2000s under the RCP 4.5 climate change scenario [45]. However, increased population and gross domestic product will lead to the water consumption increases of 15% in the 2030s if still using the current water use strategies [45]. It is noted that climate change has further exacerbated water supply in Yanchi county, as indicated by reduced precipitation and increased temperature. Mean annual precipitation in the county has declined with a rate of 0.14 mm/year, while mean annual temperature has increased with a rate of 0.02 °C/year [46]. Drought will cause more problems for the socioeconomic development of Yanchi county.

The imbalance between water supply and demand would be further exacerbated under the rural revitalization scenario. First, Yanchi government has made improvements to rural drinking water during the targeted poverty alleviation period. All residents in rural areas have gained access to safe drinking water sources. Therefore, a higher amount of rural water consumption was found from 2014 to 2019 (Figure 3b). Second, Yanchi was regarded as the demonstration area of rural revitalization in northwest China since 2019. Modern agriculture and animal husbandry development have been identified as the fundamental solution to enrich farmers. Agricultural water use increased from 6690 × 10^4^ m^3^ in 2014 to 6871 × 10^4^ m^3^ in 2019 (Figure 3b). Tan sheep breeding and related forage planting is the major source of increased water use. It is estimated that Tan sheep will be increased from the current 3.10 million to 3.80 million by 2025. However, Tan sheep production modes have changed from free grazing to house feeding since the prohibited grazing policy launched in 2002 [47]. Local government has plans to increase planting areas of day lily, Chinese medicinal materials, coarse cereals and pasture in order to provide enough forage for Tan sheep (Table 3). More water will be directly drunk by Tan sheep and will be used to irrigate forage grass. Last, improved economic levels and ecological environments also create opportunities for local tourism. Yanchi county was awarded as the demonstration area for all-for-one tourism in 2021. Tourism will increase the water demand and in particular the fresh water resources when drinking, washing, using the toilet, and maintaining the landscape [48]. 

### 4.2. Water Sustainable Use Strategy

**Figure 6:**
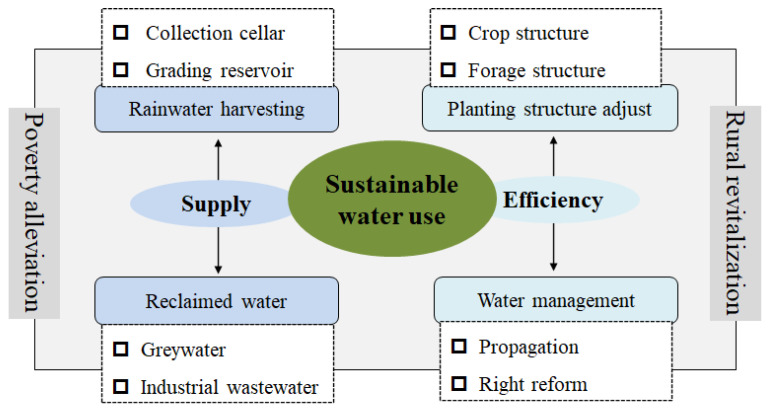
Water sustainable utilization strategies for rural revitalization in Yanchi county.

Water crisis and economic poverty are the two sustainable development issues closely related to each other [5]. Rural China, especially in the northwest region, faces the challenge of resolving the conflicts between water use and economic development. In the context of water scarcity, the coordinated configuration and efficient use of water resources are imperative during rural revitalization. In Yanchi county, water sustainable utilization can be achieved by increasing water supply from unconventional water resources and improving water use efficiency from governmental management (Figure 6). 

Development of rainwater, reclaimed urban greywater and industrial wastewater can increase water supply in Yanchi county. Rainwater collected from rooftops, courtyards and similar compacted or treated surfaces are good substitutes for rural domestic and production water [49,50,51]. Rainwater harvesting can alleviate not only water scarcity but also reduce floods in the fragile environment [12]. Collection cellars and grading reservoirs have already been designed for house and valley rainwater storage. According to the Bureau of Water Supplies of Yanchi county, total surface runoff estimation is about 2677.33 × 10^4^ m^3^, which is equivalent to the amount of groundwater. Reclaimed greywater can be used for non-potable purposes, such as irrigation, afforestation, toilet flushing and car washing [50,52]. The greywater recycling project in Yanchi county will satisfy ecological water use needs. Treated industrial wastewater can be used for various fields of production and living along with increasingly developed wastewater treatment technology [52,53]. Gaoshawo and Huianpu Industrial Park of Yanchi county is located in the ecologically fragile environment with little vegetation. Reclaimed wastewater in this area is very useful for local landscape water replenishment or the maintaining of ecological security. 

Agricultural planting structure adjustment and water-saving irrigation, and the right water reforms can improve water use efficiency in Yanchi county. The average percentage of agriculture in total water consumption was 86.15% for Yanchi county in the past six years (Figure 3b), which is higher than that of China, and on a global scale [54]. Decreasing or maintaining the water use in the agricultural sector is very important to the water sustainable use and economic sustainable development in Yanchi county. Improving agricultural water use efficiency is expected to ensure food security and sustainable water utilization [19]. A combination of degraded land consolidation engineering and modern irrigation technologies can reduce the irrigation water amount in crop growth [55]. Owing to growing crop planting area and Tan sheep feeding quantity, agricultural water requirements will increase during the rural revitalization period. However, if adopting the planting structure adjustment and water-saving irrigation technologies, reduced agricultural water demand with an amount of 6582.20 × 10^4^ m^3^ under rural revitalization scenario (Table 3) was found compared to that found in the targeted poverty alleviation period (Figure 3a). In addition, the detecting analysis results revealed that the effective irrigation area (X2), breeding stock (X4) and rural per capita net income(X5) have a significant influence on agricultural water use (Figure 4 and Figure 5). Accordingly, efficient water resource use and integrated development models of grass and livestock can coordinate ecological protection and economic development in Yanchi county [41]. For example, Tan sheep breeding involving about 80% permanent households has been proved to provide about 50% of each household’s total income. In Maerzhuang village of Fengjigou town, an automatic drip irrigation project has saved agricultural water use amounts by accurate sensing, remote transmission and intelligent analysis. The silage corn irrigation amount was decreased from four times a year with 480 m^3^ to 12 times a year with 240 m^3^. The saving water permits provide more planting areas increased from 26.7 ha to 160 ha. A high-efficiency water-saving irrigation cooperative provides the enough forage for increased Tan sheep breeding under water scarcity conditions, and increases the disposable income of farmers and the collective income of the village. This also provide the evidence to eliminate the higher interaction explanation of breeding stock (X4), rural per capita net income(X5) and effective irrigation area (X2) to agricultural water use (Figure 5). We suggested that Yanchi government establish plans for agricultural planting structure adjustment and efficient water-saving irrigation technologies for reducing areas of water-intensive crops and flood irrigation. In particular, agricultural water-saving demonstration areas should be gradually constructed in the Yellow River Irrigation District and Well Irrigation District.

The water consumption per ten thousand yuan in Yanchi county (444 m^3^) is significantly higher than that in northwest China (183 m^3^) [21]. Integrated water management measures, including governmental legislation, water-saving propagation and water right reform are suggested to address the water crisis in Yanchi county. Relevant sectors and agencies must coordinate to implement water-related laws and policies. Bottom-up initiatives should be encouraged by changing people’s values, attitudes, and behaviors toward water. In addition, water markets have played an important role in reducing the impacts of water scarcity by transferring water to its highest-valued uses [56]. Water-use quotas can allocate at various levels, including town, village, group and household. Water pricing reforms will increase people’s water-saving awareness and discourage higher water utilization. In the Yellow River Irrigation District, “Branch-cooperation-household” mode has been popularized to improve water use efficiency by synchronized tillage, planting, irrigation, fertilization and harvest. In the Yellow River Supplement Irrigation District, about 1.5 × 10^4^ ha farmland with replenishing water quota of 825 m^3^/ha was transferred to 25 agricultural companies. Agricultural irrigation can be managed according to the differences in location, industry and water sources. In the Well Irrigation District, wells were entrusted to the rural elite for management and maintenance. Water conservancy projects will save about 300 × 10^4^ m^3^ water from reducing canal leakage and evaporation from the Yellow River Diversion. 

This study only provides the sustainable use strategies of scarce water resources for rural revitalization. The specific areas and related technologies of rainwater harvesting, and the necessary responses of climate change will need to be explored in the future.

## 5. Conclusions

Northwestern arid regions of China are the key areas of ecological civilization construction and rural revitalization due to the scarce water resources and backward economic development. Rural revitalization in Yanchi county will not materialize without the support of sustainable water use. Water supply and demand balance faces a great challenge. The Yellow River diversion contributes 65.98–72.61% to the water withdrawals of Yanchi county. Groundwater exploitation has reached the available resource storage supply. Fixed diversion quota and limited ground water will make it difficult to satisfy the increased water demand from future agricultural and economic development. Sustainable water use strategies should be carried out from a water supply increment and water use efficiency perspective. Unconventional water resources, such as rainwater, reclaimed greywater and wastewater are important supplements to available water resources. Agricultural water consumption will remain or be slightly reduced in the context of rural revitalization, if governmental recommended planting structure adjustments, irrigation quotas and water use coefficient are applied. Integrated governmental legislation, water-saving propagation and water markets should be implemented to address the water crisis. In addition, rainwater harvesting should be focused on in key areas and with technology innovations. Water demand and sustainable use strategies for each sector should be formulated in accordance with rural revitalization planning under climate change scenarios.

## Figures and Tables

**Table 1:** Indicator system of agricultural water use in Yanchi county.

Indicator			Data Interpretation
Population pressure	X1	Total population	Number of permanent resident population
Farmland use	X2	Effective irrigation area (km^2^)	Irrigation area of farmland
	X3	Farmland output benefit (10^4^ Yuan/hm^2^)	Total production value/area
Industry development	X4	Breeding stock	Number of pig, cattle and sheep
	X5	Rural per capita net income (Yuan)	Net income/permanent resident population
	X6	Proportion of primary industry (%)	Primary industry product/Gross domestic product
Environmental change	X7	Annual precipitation (mm)	Sum of the twelve months’ precipitation
	X8	Annual average temperature (°C)	Average of the annual temperature
	X9	Actual evapotranspiration (mm)	Sum of evaporation of surface water, soil and plant

**Table 2:** Types of interaction between two independent variables.

Criterion	Types of Interaction
q(X1∩X2) < Min(q(X1), q(X2))	Nonlinear weakening
Min(q(X1), q(X2)) < q(X1∩X2) < Max(q(X1), q(X2))	Single-factor nonlinear weakening
q(X1∩X2) > Max(q(X1), q(X2))	Two-factor enhancement
q(X1∩X2) = q(X1) + q(X2)	independent
q(X1∩X2) > q(X1) + q(X2)	Nonlinear enhancement

**Table 3:** Agricultural water use estimation under rural revitalization in Yanchi county.

District	Planting Structure	Area (ha)	Quota (m^3^/ha)	Water Use Coefficient	Demand (10^4^ m^3^)
Yellow River Irrigation District	Maize	1227	2100	0.69	373.43
	Oil crops	2207	1725		551.75
	Medicinal materials	860	1950		243.04
	Protection forest	1227	1350		240.07
	Economic fruit	3060	1500		665.22
	Pasture	3673	2475		1317.49
	Sum	12,254	—	—	3391.00
Yellow River Supplement Irrigation District	Maize	980	2100	0.9	228.67
	Oil crops	1767	1725		338.68
	Medicinal materials	687	1950		148.85
	Protection forest	980	1350		147.00
	Economic fruit	2453	1500		408.83
	Alfalfa	2940	2475		808.50
	Sum	9807	—	—	2080.53
Well Irrigation District	Maize	547	2100	0.9	127.63
	Oil crops	987	1275		139.83
	Medicinal materials	380	1950		82.33
	Protection forest	547	1350		82.05
	Economic fruit	1367	1500		227.83
	Alfalfa	1640	2475		451.00
	Sum	5468	—	—	1110.67
Total		27,529	—	—	6582.20

## Data Availability

Not applicable.
